# Tuberculosis Treatment Outcomes and Factors Associated with Each of Them in a Cohort Followed Up between 2010 and 2014

**DOI:** 10.1155/2017/3974651

**Published:** 2017-12-28

**Authors:** Mayara A. Cardoso, Pedro Emmanuel A. A. do Brasil, Carolina Arana Stanis Schmaltz, Flavia M. Sant'Anna, Valeria C. Rolla

**Affiliations:** ^1^Postgraduate Program Clinical Research in Infectious Diseases, National Institute of Infectious Diseases Evandro Chagas-Fiocruz, Manguinhos, RJ, Brazil; ^2^Clinical Research Laboratory on Immunizations and Surveillance, National Institute of Infectious Diseases Evandro Chagas-Fiocruz, Manguinhos, RJ, Brazil; ^3^Clinical Research Laboratory on Mycobacteria, National Institute of Infectious Diseases Evandro Chagas-Fiocruz, Manguinhos, RJ, Brazil

## Abstract

Tuberculosis treatment has undergone recent changes in Brazil.* Objective*. To assess whether favorable outcomes on tuberculosis therapy improved in recent years.* Methods*. Retrospective observational study, based on primary data of tuberculosis patients, followed at INI-FIOCRUZ, from January 2012 to December 2014.* Results*. The outcomes observed were as follows: cure (80%), default (14%), treatment failure (5%), and death (1%). HIV infection without antiretroviral therapy [OR 0.34 (0.15–0.79)], tuberculosis diagnosis based on sputum smear [OR 0.22 (0.07–0.74)], drug use [OR 0.22 (0.11–0.46)], and/or treatment interruption due to adverse reactions [OR 0.23 (0.08–0.67)] decreased the chance of cure. Predictors of default, that is, use of noninjecting drugs [OR 3.00 (95% CL 1.31–6.88)], treatment interruption due to adverse reactions [OR 6.30 (1.81–21.95)], low schooling [OR 2.59 (2.15–5.82)], higher age [OR 0.44 (0.23–0.82)], and female gender [OR 0.28 (0.11–0.71)], reduced the chance of treatment default. Tuberculosis diagnosis based on sputum smear [OR 7.77 (1.94–31.09)] and/or arterial hypertension [OR 4.07 (1.25–13.18)] was associated with treatment failure.* Conclusion*. Mortality and default were low considering the prevalence of HIV infection; however cure was not significantly increased.

## 1. Introduction

Tuberculosis (TB) is still a challenge in developing countries. Brazil is one of the 22 countries with the highest number of TB cases and the 16th in the world in absolute number of cases [1]. In 2014, 67,966 new cases were diagnosed and 4,577 subjects died of TB in Brazil. At this year, default rate was 10,5% and cure rate was 72,8% [2].

Between 1979 and 2009, the Brazilian Ministry of Health recommended a standard first-line TB treatment with rifampicin, isoniazid, and pyrazinamide in the intensive phase [3]. At that time, Brazil was among the five countries in the world still using a 3-drug regimen for TB because primary resistance to rifampicin and isoniazid was just 1% in the country [4].

After 2009, the Brazilian Ministry of Health released an update with changes on TB treatment [4]. The first change was the inclusion of the 4th drug (ethambutol) in the intensive phase of therapy. This change was justified by an increase in rifampicin and isoniazid primary resistance detected during the preliminary analysis of the 2nd Brazilian resistance survey. The second change was the adoption of WHO prequalified four-drug and two-drug fixed-dose combination (FDC) pills, with a recommendation to use a daily dose of rifampicin 600 mg, isoniazid 300 mg, pyrazinamide 1,600 mg, and ethambutol 1,100 mg in the first two months (for people with more than 50 kg) of TB treatment, followed by rifampicin 600 mg and isoniazid 300 mg in the last 4 months, thus reducing dosages previously used. TB treatment with FDC pills was already recommended by World Health Organization (WHO) at that time, since there were evidences of a similar efficacy, sputum conversion, cure, and relapse rates, with operational and logistic advantages. Other update in Brazil was the availability of Rifabutin in the public health network, allowing TB/HIV patients requiring Protease Inhibitors (PI) based antiretroviral treatment to use a rifamycin as part of TB regimen [5].

Brazilian updates were implemented recently and studies regarding the impact of such strategies in therapeutic outcomes are scarce, do not address all the outcomes, were done with few HIV-TB patients, or were conducted with secondary data [6]. The aim of this study was to estimate TB treatment outcomes (cure, treatment default, treatment failure, and death) and to identify factors associated with each of them in a cohort of TB patients followed between 2010 and 2014.

## 2. Methods

### 2.1. Inclusion Criteria

The inclusion criteria included the following: (a) TB diagnosis (see below), (b) TB treatment fully conducted at INI/FIOCRUZ between 2010 and 2014, and (c) being 18 years old or older.

### 2.2. Exclusion Criteria

The exclusion criteria included the following: (a) death within the first 15 days of treatment, (b) treatment default during the first 30 days after treatment initiation, (c) change of TB diagnosis during treatment, (d) transfer to other health unit, or (e) unknown treatment outcome.

### 2.3. Design

This is a retrospective observational study, based on secondary data of a prospective ongoing cohort study conducted at the same site. This prospective study was approved by the Committee on Ethics in Research of INI-FIOCRUZ and a written informed consent form was assigned by all participant patients [7].

### 2.4. Definitions

#### 2.4.1. Tuberculosis Diagnosis

 Positive culture for* M. tuberculosis* or improvement of clinical signs and symptoms of TB, excluding other diseases, with or without a positive acid fast bacilli smear was an indication for a tuberculosis diagnosis.

#### 2.4.2. Treatment Failure

- Microbiological: a positive sputum acid fast smear or culture at 5th month of TB treatment or later.
- Clinical: symptoms and signs of TB present at the 5th month of TB treatment and radiologic evidence of TB activity

#### 2.4.3. Cure

A negative sputum culture or two negative sputum smears after 5 months of treatment indicated that TB was cured. In the absence of expectoration, cure was defined as clinical and radiological improvement when the patient finished therapy. In cases of extrapulmonary or pleuropulmonary TB with initially negative smears, cure was defined as treatment completion with clinical, radiological, and other laboratory tests improvement.

#### 2.4.4. Treatment Default

TB treatment for more than 60 days was discontinued. At INI, therapy was self-administered.

#### 2.4.5. Death

 Death meant death that occured because of TB.

#### 2.4.6. Relapse/Recurrence

Relapse or recurrence of disease means patients who were considered cured or had completed treatment but returned to the health service with a positive sputum smear.

#### 2.4.7. Effective Treatment

Effective treatment is the regimen used for the longest period during TB treatment, with or without rifampicin.

#### 2.4.8. Monoresistance

Monoresistance means resistance to only one of the first-line anti-TB drugs.

#### 2.4.9. Multidrug Resistance (MDR)

Multidrug resistance means resistance to at least isoniazid and rifampicin.

#### 2.4.10. Adverse Reactions

Only patients who had to suspend TB therapy because of adverse reactions grade 3 or 4 were considered in the analysis.

#### 2.4.11. Brazilian Minimum Wage

The minimum wage in Brazil is R$ 937,00 which is equivalent to US$ 284.

### 2.5. Data Sources/Measurements

The Clinical Research Laboratory on Mycobacteria maintains an open cohort of patients for TB diagnosis and treatment since 2000. There is a well-defined health care protocol including structured interviews and systematic collection of demographic, socioeconomic, clinical, and laboratory data as well as therapeutic outcomes of all patients during the visits, which are recorded in a data capture software. Visits take place at 0, 15, 30, 60, 90, 120, 150, and 180 days after the initiation of TB treatment. Patients who needed a longer treatment had visits after 180 days scheduled monthly up to one year. The same schedule was done for patients using rifamycins or not.

### 2.6. Analysis

Analysis was done with R-project software. Data were described as fractions, medians, and interquartile range (if not Gaussian) or means and standard deviation (if Gaussian). Logistic regression was fit for each one of the outcomes of interest (cure, treatment default, and treatment failure). Variables selection was performed by backward removal from the full model, with the Akaike Information Criterion (AIC) less than 0.05. Performance measures such as Brier score, area under the Receiver Operating Characteristic Curve (ROC), and *R*2 were estimated to allow goodness of fit interpretations.

## 3. Results

**Figure 1:**
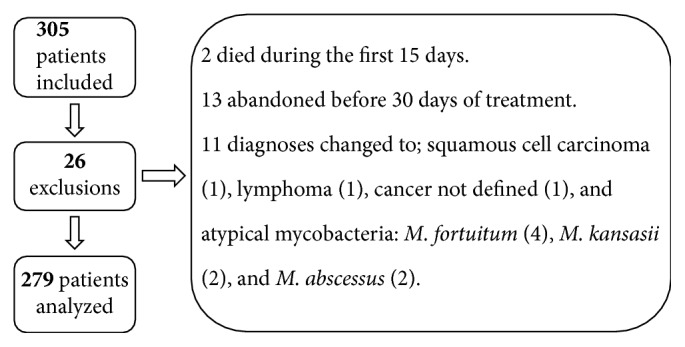
Reasons for exclusion of patients who started tuberculosis treatment at INI from 2012 to 2014.

Three hundred and five subjects were screened, but 26 were excluded, staying 279 patients in the study (Figure 1). The mean age (standard deviation) was 40.32 (15.19) years. There were just a few more men (56%) than women. The majority of patients had monthly income below tree minimum wages (70,6%). The predominant clinical form of TB was pulmonary and most of the participants attended secondary school (Table 1). HIV infection was diagnosed in 68 (24%) patients and 39 patients (57%) used antiretroviral during TB treatment (Table 2). Most of TB cases were confirmed by culture (Table 2).

### 3.1. Cure

Cure was observed in 80% of patients. The following predictors reduced the chance of cure: (a) TB diagnosis only by smear (b), noninjecting drug use, (c) TB treatment interrupted by adverse reactions, and (d) HIV+ patients without ARV therapy use during TB treatment. The logistic model showed reasonable discrimination with a ROC AUC of 0.73 and *R*2 of 0.20 (Table 3).

### 3.2. Treatment Default

The risk of treatment of default in the study period was 14%.  Table 4 shows that (a) a low education level, (b) use of noninjecting drugs, and (c) treatment interrupted by adverse reactions increased the chance of treatment default and that (d) older age and (e) female sex reduced this chance. The logistic model showed good discrimination with a ROC AUC of 0.81 and an *R*2 of 0.29.

### 3.3. Treatment Failure

The rate of treatment failure was 5%. Patients with (a) TB confirmed only by smear or (b) systemic hypertension had an increased chance of treatment failure (Table 5). The logistic model for treatment failure showed reasonable to poor discrimination with a ROC AUC of 0.65 and an *R*2 of 0.15.

### 3.4. Death

The risk of death in the study period was 1%. Three patients died and all of them were HIV positive. It was not possible to explore potential death predictors due to the very low number of deaths.

## 4. Discussion

The main results related to TB treatment outcomes are discussed below.

### 4.1. Mortality

Our study showed a low TB mortality considering that we had HIV-infected patients among TB cases. A study was conducted previously in the same site with a mortality of 6% [8]. A higher number of patients treated with HAART and TB regimens including rifamycins could explain this favorable outcome in the study period. Also, in the last 10 years, there were more social programs like Brazilian cash transfer programme (Bolsa Família Programme, BFP) in Brazilian states, including Rio de Janeiro [9]. BFP has two main objectives: transferring incomes to poor families and improving access to education and health care. Families with a monthly per capita income below US$70 are able to receive the benefit. This programme was associated with a reduction in TB incidence rate [10] and improved TB cure rate in Brazil [11]. Therefore, this cash transfer programme could have contributed to a mortality decrease observed in the present study.

A recent study performed in basic health units in Rio de Janeiro and Manaus to evaluate the performance of Xpert MTB/RIF detected a low TB attributable mortality when TB diagnosis was done by Xpert MTB/RIF instead of sputum smear [12]. We found a higher chance of treatment failure when TB diagnosis was based only on the smear results. This exam does not allow mycobacteria identification and drug sensitivity test performance which could result in misdiagnosis and makes treatment adequacy for microbiological resistance impossible. The adoption of Xpert MTB/RIF in the public network will allow MTB identification and, in addition, early detection of rifampicin resistance. This will probably increase the number of accurate TB diagnoses, which could decrease mortality associated with the disease.

### 4.2. Cure

TB diagnosis only by a positive smear was associated with another unfavorable outcome, a decreased chance of cure. These patients could have a disease caused by other non-TB mycobacteria or resistant MTB, thus leading to an inadequate treatment. Noninjectable drug users and patients with adverse reactions also had a lower chance of TB cure, probably because of a high default rate. HIV patients without ARV use during TB treatment had a higher mortality, therefore a worst cure rate.

### 4.3. Default

Lifestyle is a very important issue in the modern world and drug use is a choice made by some patients. Recently, illicit crack (cigarettes) commercialization started in Brazilian states. As it is not very expensive and a highly addictive drug, it became widespread throughout the biggest cities. In our study, noninjectable drug users were associated with therapy default. However, it was classified as a group, without the information of what type of drug was used. In a previous period, when crack was less available in Brazil, noninjectable drug users were not associated with treatment default. Male gender and younger age were also predictors of low adherence. This was also found in a study of factors influencing TB treatment default conducted in Africa [13]. Therefore, efforts should be concentrated in men and young patients to achieve a better treatment adherence since default is the worst problem when the aim is to achieve TB cure.

### 4.4. Treatment Failure

Evidence regarding the impact of FDC on treatment failure is scarce. However, a meta-analysis pointed out that FDC is not superior compared to individualized TB drugs administration to prevent treatment failure or disease relapse [14]. Another study published after this meta-analysis also suggested that FDC regimen may have performed slightly less well than separated drugs [15]. Additionally, FDC adopts lower dosages of drugs when compared to individualized formulations recommended by previous Brazilian guidelines [4]. It is possible that FDC and failure are not directly related, since the motivation for FDC adoption in Brazil was the inclusion of a 4th drug (ethambutol) to optimize TB treatment because MTB rifampicin primary resistance had raised. Another explanation already pointed out that the meta-analysis could be a reduced bioavailability of FDC pills when compared to individualized TB drugs.

In our study, a few patients failed TB treatment and most of them had TB diagnosis based only on smear results (no culture available). These could represent cases of non-TB mycobacteria diseases or MTB resistant to one or more drugs used. Those cases could have been diagnosed by rapid tests which are able to identify MTB and to give rifampicin resistance result allowing a more effective TB therapy.

Systemic hypertension was another factor associated with treatment failure and it is not explained by the condition itself. Nevertheless, this is the first study to our knowledge that reports systemic hypertension as a risk factor for failure. Further studies have to be done to better understand this result.

### 4.5. Limitations of the Study

Mortality was too low to explore potential predictors of death; however, death decrease is an important information to be considered along with recent studies performed in Rio de Janeiro that also reported lower TB mortality.

Another important point that should be considered is that INI is a peculiar health care unit, with several particular characteristics which are not representative of the whole Brazilian Health System in terms of HIV prevalence, diagnostic resources, standard protocols, and data collection.

## 5. Conclusion

Mortality and treatment default improved in recent years but this was not associated with an increased cure rate. Failure is still significant and associated with no MTB identification and systemic hypertension, which should be confirmed in further studies. Females, older subjects, and nondrug users are expected to have better outcomes, as patients with TB are diagnosed by culture and without hypertension.

Actions directed to improve treatment default have to be addressed including programs to noninjectable drug young men users. Hypertension programs could investigate possible drug interactions in patients treating for both diseases to find possible causes of TB treatment failure in hypertensive patients.

## Figures and Tables

**Table 1:** Sociodemographic and economic characteristics by outcome of patients with tuberculosis treated at INI from 2012 to 2014.

Variables	Cure	Treatment failure	Default	Dead	Total
Total	222 (80)	14 (5)	40 (14)	3 (1)	279
*Age *					
Median (IQR^a^)	38.0 (28.0–50.0)	48.0 (42.0–62.0)	32.0 (25.8–45.0)	30.0 (25.5–33.0)	38.0 (28.0–49.8)
*Sex*					
Female	108 (88)	7 (5)	7 (5)	1 (0)	123 (100)
Male	114 (73)	7 (5)	33 (21)	2 (1)	156 (100)
*Race*					
White	131 (86)	7 (4)	13 (8)	1 (0)	152 (100)
No white	91 (71)	7 (5)	27 (21)	2 (2)	127 (100)
*Schooling*					
Primary school	82 (72)	6 (5)	25 (22)	1 (1)	114 (100)
Secondary school	98 (84)	5 (4)	13 (11)	1 (1)	117 (100)
University	39 (91)	3 (7)	1 (2)	0 (0)	43 (100)
*Marital status*					
Single	105 (77)	8 (42)	24 (17)	2 (1)	136 (100)
Married	108 (81)	11 (57)	15 (11)	1 (0)	133 (100)
Widow	5 (2)	0 (0)	0 (0)	5 (1)	10 (100)
*Income* ^*b*^					
0–2	150 (76)	12 (6)	32 (16)	3 (2)	197 (100)
3–5	35 (82)	1 (2)	7 (16)	0 (0)	43 (100)
>5	20 (95)	1 (5)	0 (0)	0 (0)	21 (100)
Ignored	17 (95)	0 (0)	1 (5)	0 (0)	18 (100)
*Number of rooms*					
Median (IQR^a^)	4.0 (3.0–5.0)	4.0 (3.0–4.8)	4.0 (3.0–5.0)	3.0 (3.0–3.5)	4.0 (3.0–5.0)
*Number of residents*					
Median (IQR^a^)	2.5 (1.0–3.0)	1.0 (0.2–3.0)	3.0 (2.0–4.0)	3.0 (2.0–4.0)	3.0 (1.0–4.0)
*Homeless*					
No	218 (81)	14 (5)	34 (13)	3 (1)	269 (100)
Yes	3 (33)	0 (0)	6 (67)	0 (0)	9 (100)
*Smoker*					
No	183 (84)	8 (4)	24 (11)	2 (1)	217 (100)
Yes	38 (62)	6 (10)	16 (26)	1 (2)	61 (100)
*Smoker in the past*					
No	141 (85)	6 (4)	19 (11)	0 (0)	166 (100)
Yes	80 (71)	8 (7)	21 (19)	3 (3)	112 (100)
*Alcohol use*					
No	178 (82)	12 (6)	24 (11)	2 (1)	216 (100)
Yes	42 (69)	2 (3)	16 (26)	1 (2)	61 (100)
*Sexual behavior*					
Heterosexual	203 (81)	12 (5)	34 (13)	2 (1)	251 (100)
Homosexual/bisexual	19 (70)	2 (7)	5 (19)	1 (4)	27 (100)
*Drug use *					
No	195 (85)	13 (6)	21 (9)	0 (0)	229 (100)
Yes, *noninjecting*	27 (54)	1 (2)	19 (38)	3 (6)	50 (100)

^a^Interquartile range; ^b^values expressed in Brazilian minimum wage.

**Table 2:** Clinical forms of tuberculosis and comorbidities by outcome of patients with tuberculosis treated at INI from 2012 to 2014.

Variables	Cure	Treatment failure	Default	Dead	Total
Total	222	14	40	3	279
*Arterial hypertension*					
No	187 (80)	8 (03)	37 (16)	3 (01)	235 (100)
Yes	32 (80)	6 (15)	2 (05)	0 (00)	40 (100)
*Diabetes*					
No	198 (79)	14 (06)	37 (15)	3 (01)	252 (100)
Yes	20 (87)	0 (00)	3 (13)	0 (00)	23 (100)
*COPD* ^a^					
No	216 (79)	14 (5)	40 (15)	3 (1)	273 (100)
Yes	2 (100)	0 (0)	0 (0)	0 (0)	2 (100)
*Hepatitis B*					
No	217 (79)	14 (5)	40 (15)	3 (1)	274 (100)
Yes	2 (100)	0 (0)	0 (0)	0 (0)	2 (100)
*Hepatitis C*					
No	218 (79)	13 (5)	40 (15)	3 (1)	274 (100)
Yes	1 (50)	1 (50)	0 (0)	0 (0)	2 (100)
*HIV*					
No	171 (85)	10 (5)	21 (10)	0 (0)	202 (100)
Yes, without ARV^b^	19 (66)	2 (7)	7 (24)	1 (3)	29 (100)
Yes, with ARV	28 (72)	2 (5)	7 (18)	2 (5)	39 (100)
*Clinical form*					
Disseminated	30 (73)	3 (7)	7 (17)	1 (3)	41 (100)
Extrapulmonary	78 (87)	5 (5)	6 (7)	1 (1)	90 (100)
Pleuropulmonary	114 (77)	6 (4)	27 (18)	1 (1)	148 (100)
*Smear diagnosis*					
Negative	106 (85)	7 (6)	11 (9)	0 (0)	124 (100)
Positive	96 (72)	7 (5)	27 (20)	3 (2)	133 (100)
*Positive MTB* ^*c*^ * culture diagnosis*					
No	86 (81)	10 (9)	10 (9)	0 (0)	106 (100)
Yes	122 (78)	4 (2)	28 (18)	3 (2)	157 (100)

^a^Chronic obstructive pulmonary disease; ^b^antiretroviral; ^c^*Mycobacterium tuberculosis*.

**Table 3:** Adjusted odds ratios for risk factors for tuberculosis cure of patients treated at INI from 2012 to 2014 (ROC AUC: 0.734; *R*: 0.203; Brier: 0.138).

	Odds ratios	CI 0.95
TB confirmed only by smear	0.221	0.066–0.737
Drug use (noninjecting)	0.22	0.106–0.457
TB treatment interrupted by side effects	0.228	0.078–0.668
HIV positive without ARV use^*∗*^	0.342	0.147–0.793
HIV positive with ARV use^*∗*^	0.652	0.275–1.544

^*∗*^Baseline category: HIV negative; CI: confidence interval.

**Table 4:** Adjusted odds ratios for risk factors for tuberculosis treatment defaultof patients treated for tuberculosis from 2012 to 2014 (ROC AUC: 0.810; *R*: 0.291; Brier: 0.096).

	Odds Ratios	CI 0.95
Age	0.436	0.231–0.823
Female	0.275	0.106–0.715
Schooling, until completing elementary^*∗*^	2.589	1.152–5.821
Schooling, until completing college^*∗*^	0.379	0.046–3.119
Drug use (noninjecting)	3	1.308–6.884
TB treatment interrupted by side effects	6.3	1.809–21.948

^*∗*^Baseline category: until complete high school; CI: confidence interval.

**Table 5:** Adjusted odds ratios for risk factors for treatment failure of patients treated at INI from 2012 to 2014 (ROC AUC: 0.653; *R*: 0.150; Brier: 0.041).

	Odds ratios	CI 0.95
TB confirmed only by smear	7.775	1.945–31.087
Systemic hypertension	4.065	1.254–13.176

CI: confidence interval.

## Additional Points

*Main Contributions of the Study.* (a) Tuberculosis (TB) mortality was low between 2010 and 2014. This result was attributed to an increased number of patients on TB treatment and a more frequent HAART use during TB treatment in HIV patients. These changes coincided with Rifabutin adoption to allow different Protease Inhibitors use concomitant to TB treatment and the adoption of RHZE in fixed-dose combination (FDC) pills for tuberculosis treatment as recommended by WHO. This was also a period of government interventions, including social programs as “Bolsa Família,” particularly in Rio de Janeiro. (b) Nevertheless, the decrease in death and treatment default rates were followed by an increase in treatment failure. Higher failure risk was observed in patients who had systemic hypertension and those with TB diagnosis based only on acid fast smear (without culture results), which could represent* Mycobacterium tuberculosis* misdiagnosis or a microbiological resistance not detected. This result shows how it is important to have a TB diagnosis confirmation with sensitivity test done. (c) Adverse reactions were associated with therapy default and noninjectable drug users became an important risk group for treatment abandonment, not observed in a previous study conducted by our group. Crack recently entered in Brazil and became a new challenge. New approaches and innovative actions are being done; however, drug addiction is difficult to treat, is predisposed to TB treatment default, and allows* Mycobacterium tuberculosis *propagation.
